# Ag nanocomposite hydrogels with immune and regenerative microenvironment regulation promote scarless healing of infected wounds

**DOI:** 10.1186/s12951-023-02209-2

**Published:** 2023-11-19

**Authors:** Yihui Zhang, Jian Kang, Xuan Chen, Wenkai Zhang, Xiangqi Zhang, Wei Yu, Wei-En Yuan

**Affiliations:** 1 Shanghai Frontiers Science Center of Drug Target Identification and Delivery, School of Pharmaceutical Sciences, Shanghai Jiao Tong University, Shanghai, 200240 China; 2 Engineering Research Center of Cell & Therapeutic Antibody, Ministry of Education, and School of Pharmacy, Shanghai Jiao Tong University, Shanghai, 200240 China; 3 National Key Laboratory of Innovative Immunotherapy, Shanghai Jiao Tong University, Shanghai, 200240 China; 4 Inner Mongolia Research Institute of Shanghai Jiao Tong University, Hohhot, China

**Keywords:** Ag nanoparticles, Nanocomposite hydrogel, Immune and microenvironment regulation, Wound healing

## Abstract

**Background:**

Bacterial infection, complex wound microenvironment and persistent inflammation cause delayed wound healing and scar formation, thereby disrupting the normal function and appearance of skin tissue, which is one of the most problematic clinical issues. Although Ag NPs have a strong antibacterial effect, they tend to oxidize and form aggregates in aqueous solution, which reduces their antibacterial efficacy and increases their toxicity to tissues and organs. Current research on scar treatment is limited and mainly relies on growth factors and drugs to reduce inflammation and scar tissue formation. Therefore, there is a need to develop methods that effectively combine drug delivery, antimicrobial and anti-inflammatory agents to modulate the wound microenvironment, promote wound healing, and prevent skin scarring.

**Results:**

Herein, we developed an innovative Ag nanocomposite hydrogel (Ag NCH) by incorporating Ag nanoparticles (Ag NPs) into a matrix formed by linking catechol-modified hyaluronic acid (HA-CA) with 4-arm PEG-SH. The Ag NPs serve dual functions: they act as reservoirs for releasing Ag/Ag^+^ at the wound site to combat bacterial infections, and they also function as cross-linkers to ensure the sustained release of basic fibroblast growth factor (bFGF). The potent antibacterial effect of the Ag NPs embedded in the hydrogel against *S.aureus* was validated through comprehensive in vitro and in vivo analyses. The microstructural analysis of the hydrogels and the in vitro release studies confirmed that the Ag NCH possesses smaller pore sizes and facilitates a slower, more sustained release of bFGF. When applied to acute and infected wound sites, the Ag NCH demonstrated remarkable capabilities in reshaping the immune and regenerative microenvironment. It induced a shift from M1 to M2 macrophage polarization, down-regulated the expression of pro-inflammatory factors such as IL-6 and TNF-α, and up-regulated the expression of anti-inflammatory IL-10. Furthermore, the Ag NCH played a crucial role in regulating collagen deposition and alignment, promoting the formation of mature blood vessels, and significantly enhancing tissue reconstruction and scarless wound healing processes.

**Conclusions:**

We think the designed Ag NCH can provide a promising therapeutic strategy for clinical applications in scarless wound healing and antibacterial therapy.

**Graphical Abstract:**

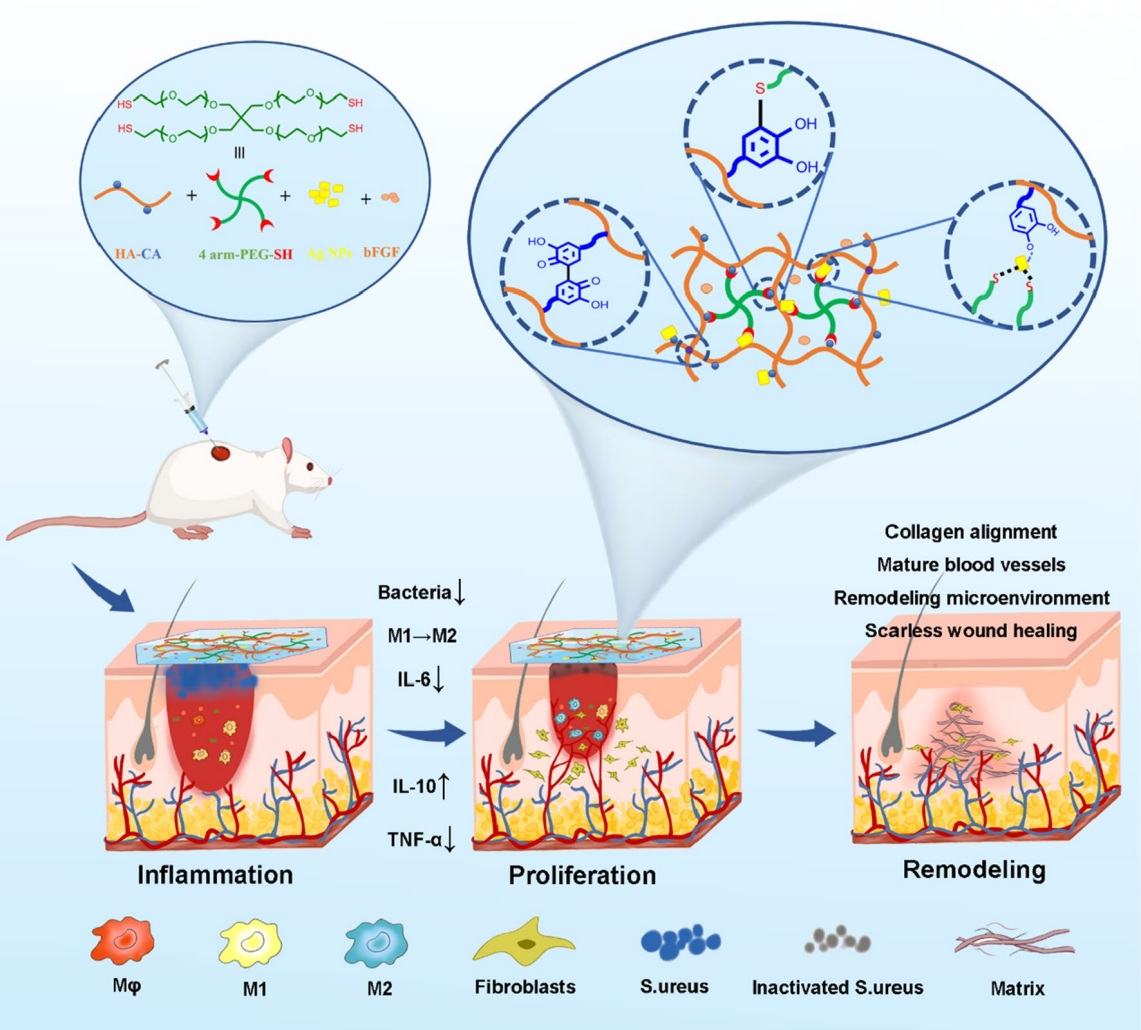

**Supplementary Information:**

The online version contains supplementary material available at 10.1186/s12951-023-02209-2.

## Background

Skin is the first line of defense against external invasion, protecting the body from chemical and physical damages and invasion by pathogens [1]. However, the structure and function of the skin can be damaged to varying degrees following surgery, burns, or other injuries. Generally, wound healing is a complex, dynamic and sequential biological process, consisting of three overlapping stages of hemostasis and inflammation, proliferation and remodeling [2]. However, this process can be disrupted by bacterial infection, inflammation, and growth factor inactivation, which can result in delayed healing, scarring, or even systemic inflammatory response syndrome (SIRS), a potentially life-threatening condition [3].

Scarring is a result of excessive fibroblast activity and abnormal collagen deposition, which impair tissue function and appearance [4]. Wound healing that leads to scarring involves a sequence of events, starting with local inflammation. Excessive inflammation results in abnormal production of growth factors, proteolytic enzymes, angiogenic factors, and fibrotic cytokines, which together stimulate the deposition of connective tissue and alter the normal tissue structure [5, 6]. During this process, uncontrolled activation of macrophages affects the quality of wound healing. Macrophages in a state of unrestricted pro-inflammatory M1 activation and incomplete conversion to M2 secrete high levels of TNF-α and hydroxyl radicals, resulting in failure of chronic venous ulcers to heal [7, 8]. Recent work has utilized the anti-inflammatory state to shorten the inflammatory phase and thus promote healing and repair [9, 10]. The environment of the wound site, such as infection, hypoxia, increased lactic acid and free radical production, is also closely associated with the development of scars.

The wound is exposed to bacterial invasion immediately after injury due to the loss of the skin as a protective layer. Bacterial colonization of the wound site can prolong the inflammatory period, increase the uptake of macrophages, and decrease the healing effect [11–13]. Ag NPs have strong antibacterial effects and accelerate cell proliferation by reducing cellular inflammation and promoting epithelial re-formation [14]. However, Ag NPs have low stability in solution, are easy to oxidize, and tend to form aggregates. This results in reduced antibacterial efficacy at certain concentrations and potential toxicity to tissues and organs. This problem is solved by mixing Ag NPs into biocompatible carriers such as hydrogels to form NCH [15]. NCH is a physical or covalent cross-linked polymer network with a 3D structure containing covalent or non-covalent fixed nanoparticles or nanostructures in the matrix [16]. They can be used for the delivery of drugs and bioactive molecules, as warehouses or vectors for targeted and intelligent release, etc [17, 18].

Research on scar treatment is limited and currently focuses on the use of growth factors and drugs to reduce inflammation and limit scar tissue formation [19, 20]. Basic fibroblast growth factor (bFGF) expression is increased in acute wounds and mediates wound healing and scar formation by regulating cellular behavior, including influencing cellular chemotaxis, stimulating angiogenesis, and extracellular matrix (ECM) synthesis and degradation, which is a key regulator of wound healing and tissue repair [21]. With the development of recombinant growth factors, the short life span of these molecules is no longer a major challenge. However, there is still a need for better delivery systems to modulate the complex wound microenvironment, to administer drugs in a controlled manner and to reduce the frequency of administration [22, 23]. HA is a non-sulfated linear glycosaminoglycan polymer composed of repeating disaccharide units of N-acetyl-d-glucosamine and d-glucuronic acid. HA has physicochemical properties such as water retention, hygroscopicity, rheology, and viscoelasticity [24]. HA acts as a scaffold to assemble the extracellular matrix, and the space created by HA helps inflammatory cells and fibroblasts infiltrate the wound site [25]. Besides, fetuses are known to have scarless repair capabilities due to the high concentrations of HA in fetal ECM [26].

Herein, we constructed the Ag NCH to modulate the immune and wound microenvironment for scarless wound healing of infected wounds. Benefiting from abundant CA groups, biomaterial matrix and the fixed Ag NPs, the Ag NCH has the advantages of adjustable pore size, superior biocompatibility and antibacterial properties. Ag NPs were immobilized in the composite system as cross-linkers, avoiding high cytotoxicity associated with direct contact with cells and enhancing the network structure. The Ag NCH is capable of releasing Ag/Ag^+^ and bFGF to the wound site as needed. In vivo experiments have demonstrated that it promotes macrophage polarization, shortens the inflammatory process, regulates collagen deposition, and promotes the formation of mature blood vessels. These results indicate that Ag NCH can remodel the immune and regenerative microenvironment and facilitate the healing and scarless repair of acute and bacterial infected wounds.

## Methods

### Materials

1-(3-Dimethylaminopropyl)-3-ethylcarbodiimide hydrochloride (EDC), N-Hydroxy succinimide (NHS), HA (MW:40–80 kDa) were obtained from Shanghai Macklin Biochemical Co., Ltd (China). 4-arm PEG-SH (MW:2 kDa) was purchased from Shanghai Yuanyang Bio Tech. Inc. (China). Ag nanodispersion solution was purchased from Xiamen Numan Technology Co., Ltd. (China). bFGF (35000 IU) was purchased from Beijing SL Pharmaceutical Co., Ltd. (China). Dopamine hydrochloride (DA∙HCl) were purchased from Sigma-Aldrich (St. Louis, MO, USA). CCK-8 was purchased from Dojindo Molecular Technologies, Inc. (Japan). Mouse Embryonic Fibroblast Cells (NIH/3T3) were ordered from the cell bank of Chinese Academy of Sciences (Shanghai, China). All the antibodies were purchased from Abcam (China).

### Synthesis and structural characterization of HA-CA conjugates

HA-CA conjugates were synthesized by EDC/NHS coupling reaction as described by Zhong et al. [27]. Specifically, a 0.5% (w/v) solution of HA was prepared by dissolving HA in deionized water, and then NHS and EDC with molar ratios of 1.5:1 and 1:1 to HA were added. After stirring for 1 h, DA·HCl was added at a molar ratio of 1.5:1 (DA·HCl : HA). The pH of the solution was adjusted to 5.0. The reaction was conducted overnight at room temperature under nitrogen. Subsequently, the reaction solution was dialyzed against ultrapure water at a low temperature for 2 days and lyophilized, obtaining HA-CA. The HA-CA conjugates were confirmed using FTIR spectroscopy (Nicolet 6700, Thermo scientific, USA), and the degree of substitution of dopamine was determined by the ^1^H NMR spectrum (Avance III, Bruker, Germany) by calculating and comparing the integral area of the methyl proton of acetamide and CA proton in HA.

### Characterization of Ag NPs

Transmission electron microscopy (TEM, Talos L120C G2, Thermo Fisher, USA) was used to characterize the morphology of the nanoparticle. Nanoparticle size and zeta potential analyzer (Zetasizer Nano S, Malvern, UK) was used to measure the hydrodynamic size and zeta potential of nanoparticles.

### Fabrication of Ag NCH

HP hydrogels were prepared by dissolving HA-CA (1%, w/v) in 0.01 M phosphate buffer (PBS, pH = 5.8) and mixing it with 125 mg/mL 4-arm PEG-SH solution at a volume ratio of 8:1. The pH of the hydrogel was adjusted to 7 with 1 M NaOH, and the mixture was rapidly stirred at room temperature. After about 30 s, the solution was converted to hydrogel. Ag NCH was prepared by centrifuging the nanodispersion and dispersing it into PBS to obtain a 20 μg/mL (Ag_H_) nanodispersion, which was then diluted to 5 μg/mL (Ag_L_) and 10 μg/mL (Ag_M_), respectively. The nanodispersion was then mixed with HA-CA solution and 4-arm PEG-SH solution. Biologically active agents such as bFGF and Ag NPs are added to the system in the mixing stage by embedding method. Finally, the PH of the mixture was raised to 7 and stirred to form hydrogel.

### Morphology of hydrogels

Scanning electron microscopy (SEM, MIRA3, TESCAN, CZE) was used to observe the microporous structure of hydrogels. The freeze-dried samples were coated with gold for 30 s before observation. The accelerating voltage was 20 kV. About 50 pores on SEM images were analyzed by ImageJ software, and the average pore size was calculated.

### Antioxidant efficiency

The antioxidant capacity of the hydrogel was tested by measuring its ability to scavenge DPPH free radicals. The catecholyl group of HA-CA in hydrogel can remove DPPH free radical. First, the prepared HP hydrogel was homogenized and dispersed in ethanol. DPPH (3.0 mL, 100 µM) was then added and incubated in the dark for 30 min. Then, the absorbance at 518 nm was measured using a microplate reader. The DPPH free radical scavenging ability of hydrogels was evaluated using the following formula:

$$DPPH{\text{ }}scavenging{\text{ }}\% \, = \,\left( {AB - AH} \right)/{\text{ }}AB\, \times \,100.$$

Where AB is the absorption of blank (DPPH + ethanol) and AH is the absorption of hydrogel (DPPH + ethanol + hydrogel).

### in vitro bFGF release

The prepared HP/bFGF hydrogels, HP-Ag_L_/bFGF, HP-Ag_M_/bFGF, and HP-Ag_H_/bFGF hybrid hydrogels were placed in 1 mL PBS (pH = 7.4) in a constant temperature stirrer at 37 ℃ and 100 rpm. At 1 h, 6 h, 1d, 2d, 4d, 8d and 14d, 1 mL of the release solution was collected and supplemented with 1 mL of fresh PBS. The released solution was diluted to the corresponding concentration, and the protein drug content was determined by Micro BCA method.

### Bioactivity of released bFGF

The bioactivity of the released bFGF was evaluated using a proliferation assay of NIH/3T3. NIH/3T3 cell suspensions were seeded in 96-well plates at concentrations of 1.0 × 10^5^ cells/well. After incubation for 24 h, the medium was replaced with release medium supplemented with 0.5% FBS. After 48 h of culture, cell viability was assessed by CCK-8 assay. Medium with 0.5% FBS and the released medium collected from HP-Ag_M_ were used as controls. The relative cell viability was quantified by normalizing the absorbance of the release medium to the absorbance of medium with 0.5% FBS.

### in vitro Ag release

The HP-Ag_L_/bFGF, HP-Ag_M_/bFGF and HP-Ag_H_/bFGF hybrid hydrogels were immersed in 2 mL of deionized water at 37 °C. The deionized water was collected and then supplemented with fresh deionized water at predetermined intervals (1 d, 3 d, 5 d, 9d and 14 d). The amount of released Ag was quantified using inductively coupled plasma optical emission spectrometry (ICP-MS, i CAP Q, Thermo, USA). The test was repeated three times.

### Distribution of Ag NPs in the hydrogel

Ag NPs hydrogel solution with an appropriate concentration was pre-frozen in ethane for testing. The distribution of Ag NPs was observed by low temperature TEM (Talos F200C G2, FEI, USA).

### Rheological Tests

The rheological properties were monitored by dynamic rheometer (ARG2 rheometer, TA Instruments, USA). The hydrogel was loaded onto the rheometer plate, and the upper parallel plate with a 40 mm diameter was lowered to 500 μm at 25 °C to fill the gap between the bottom plate and the probe. To prevent evaporation of water, a heat shield and a humid environment are provided. For oscillatory time sweep experiments, the storage moduli (G′) was measured from 0.1–100 rad/s at 1% strain.

### Compression tests

Compression tests were performed on cylindrical hydrogels with a height of 8 mm and a diameter of 15 mm using a dynamic mechanics analyzer (DMA, Q850, TA Instruments, USA). The hydrogels were compressed to 100% strain at a constant rate of 0.5 mm/s, and stress–strain curves were drawn.

### Thermal analysis of hydrogels

The TGA curves of the hydrogels were recorded using a synchronous thermal analyzer (SDT-Q600, TA Instruments, USA) heated at a rate of 10 °C /min under nitrogen.

### Biocompatibility of hydrogels

The hydrogel extracts were co-cultured with NIH/3T3 cells to evaluate the biocompatibility of the hydrogels. Sterile hydrogels were incubated in 48-well plates at 37 °C in culture medium for specific times. The hydrogels were then removed to obtain the extraction medium. NIH/3T3 cell suspension was seeded in 48-well plates at concentrations of 1.0 × 10^5^ cells/well (cytotoxicity assay) and 5.0 × 10^3^ cells/well (proliferation assay), and incubated at 37 °C and 5% CO_2_ for 24 h. The medium was then removed and the corresponding hydrogel extract was added and incubated. The CCK-8 assay was used for the determination of cytotoxicity and proliferation.

### Hemocompatibility of Hydrogels

Hemolysis rates of HP and HP-Ag_M_ hydrogels were measured in vitro. Fresh uncoagulated blood was collected from mice and centrifuged at 250 g for 10 min to obtain red blood cells (RBCs). RBCS were diluted 50 times with 0.9% normal saline to obtain RBC stock dispersions. The hydrogel was preheated at 37 ℃ and added to 200 μL of RBC stock solution dispersion, stirred at 37 ℃ for 1 h, and centrifuged at 1800 rpm for 5 min to obtain the supernatant. The absorbance of the supernatant was measured at 545 nm (n = 3) using distilled water and PBS as positive and negative controls, respectively. Hemolysis rate was calculated using the following formula:

$$HR{\text{ }}\left( \% \right){\text{ }} = {\text{ }}\left( {Ab{s_{samples}} - {\text{ }}Ab{s_{PBS}}} \right)/\left( {Ab{s_{water}} - {\text{ }}Ab{s_{PBS}}} \right) \times {\text{ }}100\%$$

### Antibacterial activity in vitro

The antibacterial activity of HP-Ag hydrogel against *S.aureus* (ATCC 6538) strains was determined. Bacterial solution in logarithmic growth phase was diluted with PBS to 10^6^ CFU/mL. HP-Ag hydrogels were formed in situ on 24-well plates, and 1 mL of bacterial suspension was added to the surface of each hydrogel and incubated for 24 h at 37° C. Bacterial solution cocultured in PBS as a control group. The bacterial solution was diluted 10^7^ times with PBS, and 100 μL of the bacterial suspension was uniformly dispersed onto the agar plate with sterile swabs. After incubation at 37  C for 12 h, photographs of the colonies were taken. In addition, 100 μL of bacterial solution was transferred to a 96-well plate for another 12 h, and the absorbance value at 600 nm wavelength was measured by microplate reader.

### in vivo wound healing assay

Adult female Balb/c mice (6–8 weeks) were purchased from Shanghai Slack Laboratory Animal Co., LTD (Shanghai, China). The full-thickness skin defect model was established to evaluate the healing efficacy of the hydrogels. Briefly, a round skin wound with a diameter of 1 cm was produced on the back of mice using sterilizing scalpels. The mice were separated randomly into 4 groups (n = 9): control, HP hydrogel, HP/bFGF hydrogel and HP-Ag/bFGF hydrogel. A 100 μL of hydrogel solution (pH = 7) was injected into the wound and converted into hydrogels after 30 s. The control group was not treated after skin injury. The mice were sacrificed at 3, 7 and 14 days after injury.

When constructing the infected wound model, *S. aureus* droplets were diluted to 1.0 × 10^7^ CFU/mL and added to the established wound. Similarly, mice were randomly divided into 3 groups (n = 9): control group, bFGF group and HP-Ag/bFGF group. bFGF and HP-Ag/ bFGF hydrogel solution were added to the wound site 2 h later. The control group received no treatment. The mice were sacrificed at 4, 7 and 14 days after injury.

### Wound healing observation

The wound areas were photographed and measured using ImageJ sofrware. The formula for calculating the wound healing rate was as follows:

$$Wound{\text{ }}healing{\text{ }}rate{\text{ }}\left( \% \right){\text{ }} = {\text{ }}\left( {1 - {S_n}/{S_0}} \right){\text{ }} \times {\text{ }}100\%$$

where S_n_ is the wound surface area on day n and S_0_ is the wound surface area on day 0.

### Bacterial culture in infected wounds

The mice were sacrificed on day 7 and the infected skin tissue of the wound was preserved in sterile PBS at 4 ℃. A tissue homogenizer was used to homogenize the tissue, and excess tissue blocks were removed with a 200-mesh nylon screen. After the filtrate was diluted to an appropriate concentration, 100 μL was smeared in LB solid medium. After 24 h of culture in 37 ℃, the number of colonies was recorded.

### Histological analysis

Mice were euthanized using carbon dioxide inhalation. The skin tissues of the post-injury site were harvested, fixed in 4% formalin for 24 h, embedded in paraffin, and cut into 5-μm-thick sections through the wound center. Histopathology analysis was performed after hematoxylin and eosin (H&E) staining and Masson’s trichrome staining. Immunohistochemistry was used to detect IL-6, IL-10, TNF-α, VEGF, CD31, TGF-β, and α-SMA in sections. Relevant semi-quantitative analyses such as collagen deposition and the number of vascular profiles distributed in the wound bed were assessed using ImageJ. Histological images of stained sections were captured with an optical microscope (Olympus BX53, Japan).

### Immunofluorescence staining

To distinguish between pro-inflammatory M1 macrophages and anti-inflammatory M2 macrophages in the spleen, macrophage classification markers were double-stained with anti-F4/80 & anti-CD11c (M1) and anti-F4/80 & anti-CD206 (M2) (Abcam, Cambridge, United Kingdom), respectively. The immunofluorescence staining was observed with a fluorescence microscope (Olympus BX53, Japan) and the images were collected.

### Flow cytometry analysis

In parallel, M1 and M2 macrophage were characterized by flow cytometry. The spleen was pressed at the bottom of a 1 mL syringe through a 300-mesh nylon screen using the grinding method, and the prepared single-cell suspension was collected in RPMI 1640 medium. Following the manufacturer’s instructions, the cell suspensions (100  μL, 10^6^ cells/mL) after erythrocyte lysis were incubated with fluorochrome-conjugated anti-mouse anti-F4/80-FITC, anti-CD11c-PerCP and anti-CD206-APC antibodies (BioLegend, San Diego, CA, USA) at 4 °C for 30 min in the dark. After incubation, the cells were washed twice with PBS and the labeled cells were acquired on a flow cytometer (LSRFortessa, BD Biosciences). The dispersions were analyzed using the FlowJo software (BD Biosciences).

### Statistical analysis

All data are presented as mean standard deviation. Image J software was used to quantitatively process the experimental Image data. One-way ANOVA (GraphPad Prism, Inc.) was used to calculate the significance between the means. All experiments were performed in triplicate (n = 3) unless otherwise stated. P < 0.05 was considered as statistically significance (*: P < 0.05, **: P < 0.01, ***: P < 0.001, ****: P < 0.0001).

## Results and discussion

### Synthesis and formation mechanism of functionalized HA hydrogels

**Fig. 1:**
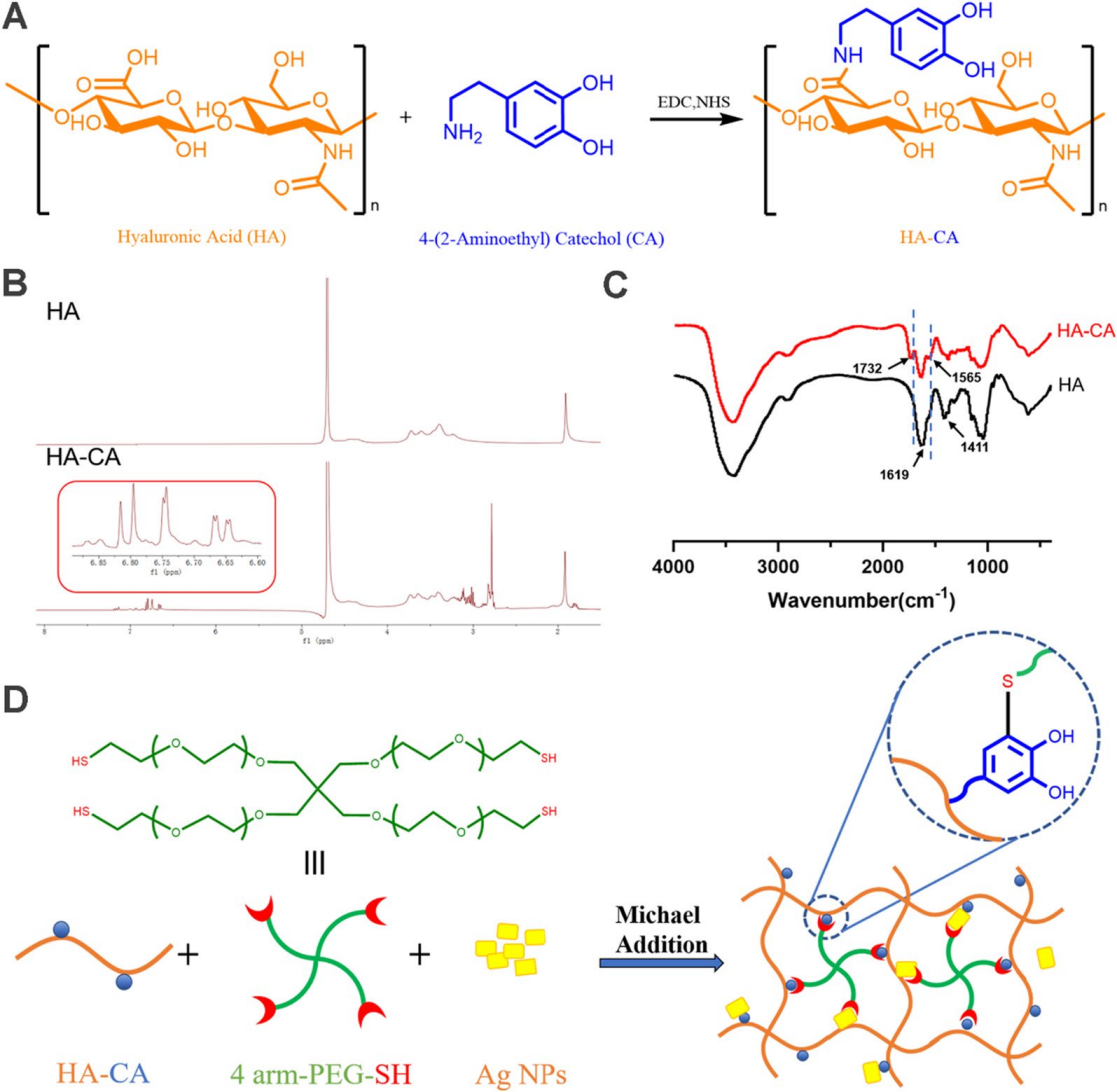
Structure and schematic of hydrogel formation. **A** Synthesis of HA-CA conjugates. **B** ^1^H NMR spectra and **C** FTIR spectrum of HA-CA conjugates. **D** Schematic of the HP-Ag structure.

As shown in Fig. 1A, CA was grafted onto HA by EDC/NHS chemistry to produce the conjugate HA-CA. The copolymer structure was validated by ^1^H NMR spectra and FTIR spectrum, as shown in Fig. 1B and C. The peak observed at δ 1.8–2.0 corresponds to the proton in methylene of HA. The conjugation of CA to HA was confirmed by the presence of aromatic-proton peaks at δ 6.70–6.85 ppm and methylene-proton peaks at δ 3.1 and 2.8 ppm. According to the calculation, the substitution degree of CA in HA was 12.9%. In comparison with the FTIR spectrum of HA, the absorption peak at 1732 cm^−1^ may assigned to the stretching vibration peak of − COOH. In addition, the asymmetric and symmetric stretching vibration intensities of − COONa at 1619 cm^−1^ and 1411 cm^−1^ were significantly reduced. Even more important, HA-CA showed a stronger absorption peak at 1565 cm^−1^, which was formed by N–H bending vibration of amide II band. These findings confirm successful conjugation between HA and CA via amide bonds. The schematic of HP-Ag structure is shown in Fig. 1D. CA loses two electrons and two protons to become quinone, which reacts highly with the mercaptan group through the Michael addition reaction. CA groups can form physical or chemical bonds with different surfaces, and quinone groups promote the cohesion of hydrogels [28].

### Physicochemical properties of the hydrogel

**Fig. 2:**
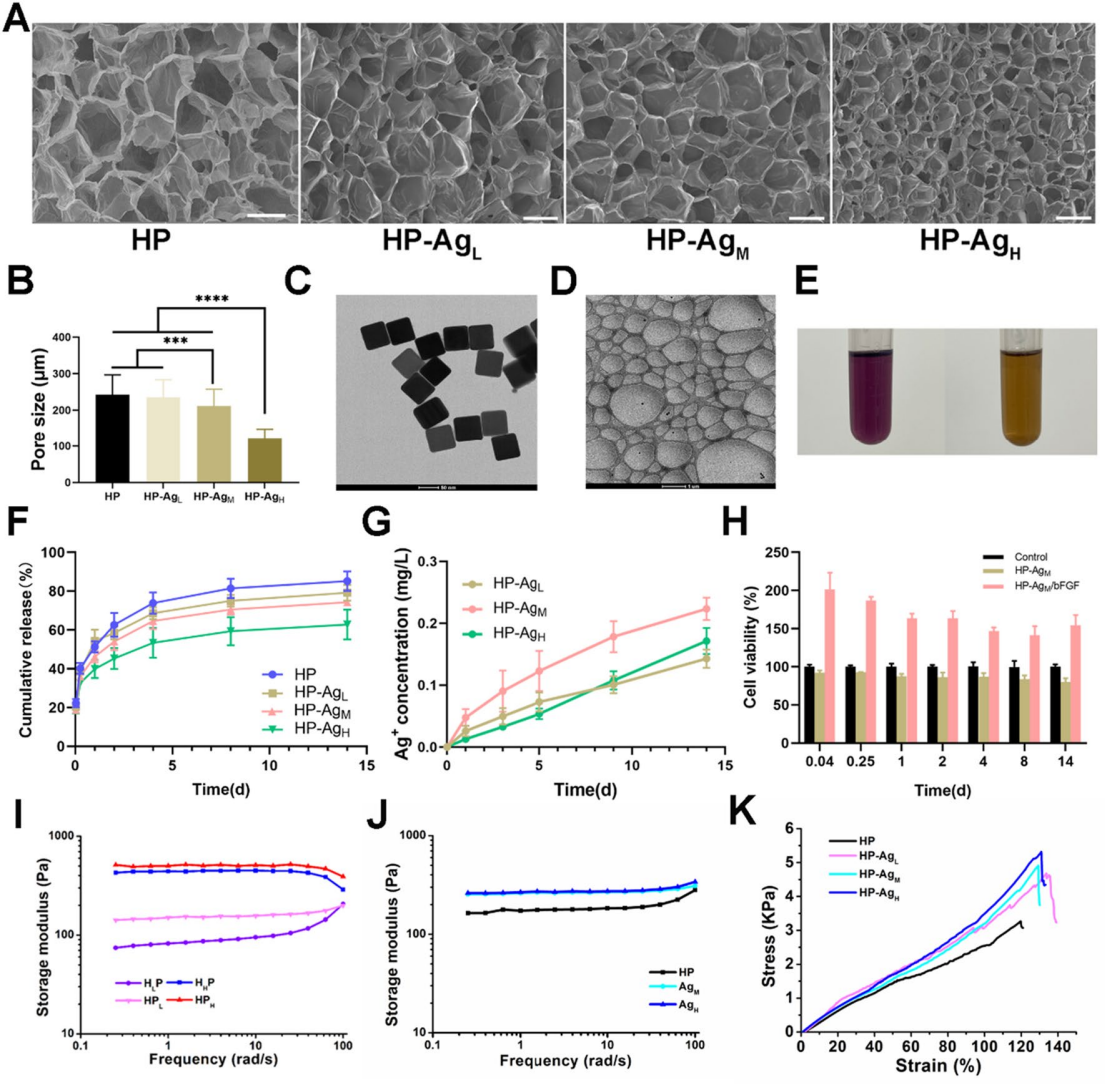
Physicochemical properties of HP-Ag/bFGF hydrogel. **A** Scanning electron microscope images of the freeze-dried HP-Ag hydrogel. Scale bar: 500 μm. **B** The pore size of the hydrogels prepared by different concentrations of Ag NPs. **C** Morphology of of Ag NPs. **D** TEM micrograph of Ag NPs in HP-Ag hydrogel. **E** DPPH radical scavenging rate of HP hydrogel. **F** Cumulative release curve of bFGF in HP-Ag/bFGF hydrogel. **G** Cumulative release curve of Ag^+^ in HP-Ag hydrogel. (**H**) Bioactivity of released bFGF. **I** G′ of HP hydrogels with different matrix contents measured in frequency sweep. **J** G′ of HP-Ag hydrogel measured in frequency sweep. **K** Compressive stress–strain curves of HP-Ag hydrogel. Data are presented as mean ± S.D, n = 3

The micromorphology and average pore size of freeze-dried hydrogels with different Ag NPs contents are shown in Fig. 2A and B. All hydrogels exhibit a uniform network structure and porous interpenetration. The crosslinking density can be reflected by hydrogel pore size to some extent. With the increase of Ag NPs content, the pore size of hydrogel gradually decreases. The average pore sizes of HP, HP-Ag_L_, HP-Ag_M_ and HP-Ag_H_ were 242 ± 54.4 μm, 234 ± 48.3 μm, 211 ± 46.2 μm and 122 ± 25.3 μm, respectively. The porous structure allows the hydrogel to absorb blood and exudate from the wound tissue, providing enough space for cell proliferation and thus speeding up hemostasis [29]. The addition of Ag NPs enhances the gel network structure and occupies the ordered pores in the hydrogel, leading to a reduction in pore size. Two possible causes of gel network enhancement are: First, CA on the HA-CA polymer deprotonates to form quinone groups, which help the deposition of HA-CA on the Ag surface through hydrophobic action; Secondly, sulfophilic Ag can react with available sulfhydryl groups in 4-arm PEG-SH to form Ag–S bond, which attach the PEG-SH to Ag NPs surface [30–32].

Ag NPs have a large specific surface area, and their antibacterial activity is affected by their particle size. From Fig. 2C and Additional file 1: Fig. S1, it can be observed that Ag NPs are in regular cube shape with a particle size of 100 nm and a potential of − 23.87.

As shown in Fig. 2E, the DPPH-clearance of the HP hydrogel was 59.53%. The clearance was lower than expected, considering that there were fewer CA groups available on HA, as some of them may have been oxidized to quinone or attached to other groups.

The release behavior of bFGF in HP-Ag/bFGF was studied, and it can be clearly observed from Fig. 2F that bFGF was released rapidly in HP hydrogel, while the incorporation of Ag NPs slowed down the release rate. For HP hydrogel, a burst release of bFGF (51.3%) was observed on day 1, while 46.2% and 39.9% bFGF release were observed in HP-Ag_M_ and HP-AgH hydrogels, respectively. The release in HP hydrogel reached 73.9% on day 4, and the release rate in HP-Ag_M_ and HP-Ag_H_ hydrogels were 64.4% and 53.4%, respectively. These pieces of evidence suggest that long-term stable release can be achieved by adding Ag NPs to the hydrogel to enhance the network structure, thus simplifying drug administration.

The antibacterial activity of HP-Ag hydrogel depends on the release of Ag from the hydrogel into the infected site. Ag NPs can not only cross-link networks in hydrogel, but also act as reservoirs for Ag. As shown in Fig. 2G, a steady and sustained release of Ag was observed throughout the test time. The quantitative method of Ag release used does not allow the differentiation of its form (ion or nanoparticle) [33]. The cumulative Ag releases of the HP-Ag_L_, HP-Ag_M_ and HP-Ag_H_ after 14 days were 0.143 mg L^−1^, 0.203 mg L^−1^ and 0.171 mg L^−1^, respectively. The relatively high release on the first day ensured a strong antibacterial effect during the initial phase of tissue healing, and a certain amount of Ag remained at the wound site although the release of Ag was reduced later [34]. The current release was slightly lower than expected [35–37], possibly because some Ag NPs participated in the network as a crosslinking agent, and the surface of Ag NPs was attached to HA-CA or 4-arm PEG-SH, thus delaying Ag release. This low concentration ensures effective sterilization above subinhibitory concentrations to avoid the formation of bacterial biofilms, while being safe for cells and tissues [32, 33, 36].

In general, Ag NPs tend to accumulate and can lead to large increases in local Ag concentrations, which may affect cell activity. The result shows that Ag NPs were distributed uniformly in HP-Ag (Fig. 2D). It is speculated that the hydroxyl and sulfhydryl groups deposited on the surface of Ag NPs cause the stable and uniform distribution of Ag NPs in the network structure.

The bioactivity of released bFGF was evaluated by the stimulating effect on NIH/3T3 cell viability. From Fig. 2H, compared with maintenance medium group and HP-Ag group, the cell viability of the bFGF release group was significantly increased, indicating that bFGF in hydrogel maintained bioactive.

The dynamic rheological analysis was conducted to investigate the mechanical performance of hydrogels. Figure 2I and J shows the storage modulus (G′) of hydrogels with different contents of HA-CA, 4-arm PEG-SH and Ag NPs measured in frequency sweep. All hydrogels showed elastic solid-like hydrogel after gelation. The G' of hydrogel increased with the increasing concentration of HA-CA and 4-arm PEG-SH. Due to the introduction of HA-CA and 4-arm PEG-SH, the network cross-linking of hydrogels was enhanced through multiple interactions, and the stiffness of hydrogels was improved. Similarly, the G' of HP-Ag hydrogel gradually increased with the increase of Ag NPs content. The G' increased with the increase of strain frequency within the angular frequency of 0.1–100 rad s^−1^. The increase in modulus with strain frequency is the strain-stiffening behavior of hydrogels[38]. This property enables hydrogel to produce a better bonding effect when high strain and strong stress are generated at the bonding site[39].

Compressive stress–strain tests were performed on the hydrogels to assess its mechanical strength (Fig. 2K). Consistent with the rheological results, the stresses of HP-Ag/bFGF hydrogels were higher than that of HP hydrogel. Specifically, the compressive strength of HP hydrogel was as low as 3.3 kPa, while the compressive strength of HP-Ag_L_, HP-Ag_M_ and HP-Ag_H_ increased by 4.7 kPa, 4.9 kPa and 5.3 kPa, respectively. With the increase content of Ag NPs, the compressive rupture strain of hydrogel also increased slightly. The compressive rupture strain of HP hydrogel was 119.7%, and that of HP-Ag_L_, HP-Ag_M_ and HP-Ag_H_ were 133.6%, 129.3% and 130.9%, respectively. From Fig.S2A, the HP hydrogel has an additional weight loss temperature range compared to the weight loss of HA, with complete thermal decomposition occurring at the 300–420 °C stage. From Fig.S2B, the total residual weight of HP-Ag_H_ was always higher than that of the HP-Ag_L_ composite system at each weight loss stage. These results indicate that Ag NPs are embedded in the hydrogel matrix and the hydrogels with higher Ag NPs content can form more stable structures. In summary, these results indicate that HP-Ag/bFGF hydrogel holds several merits including porous microstructure, bFGF release regulation, Ag release and adjustable stiffness capability.

### in vitro biocompatibility and antibacterial activity

**Fig. 3:**
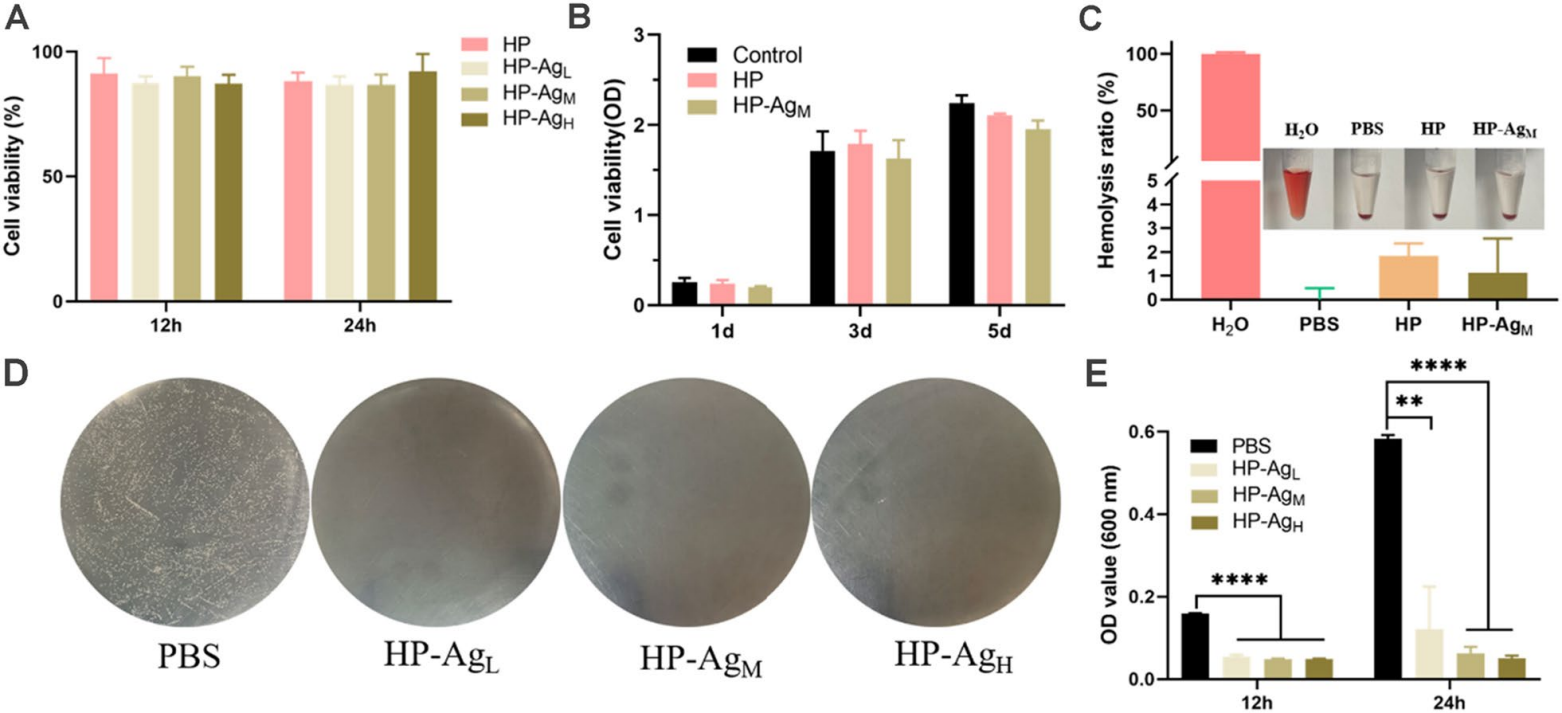
Biocompatibility and antibacterial properties of hydrogels. Cell viability of NIH/3T3 co-cultured with hydrogel extracts for **A** 12 h and 24 h. and **B** 1d, 3d and 5d. **C** Hemolytic activity assay of HP and HP-Ag_M_ hydrogel. Inset is the corresponding photograph. **D** Photographs of survival bacteria colonies (*S. aureus*, white dots in plates) growing on agar plates after direct contact incubation with HP and HP-Ag hydrogels. PBS was used as control. **E** OD values at 600 nm of S. aureus solution co-cultured with the hydrogels for 12 h and 24 h. All data are presented as mean ± SD, n = 4, **p < 0.01, ***p < 0.001, ****p < 0.0001

The versatility of hydrogels inspired us to explore the biological properties required for their application in wound dressings. Good biocompatibility is an essential prerequisite for hydrogel dressings because they are in direct contact with tissues and blood in practical applications. The cytotoxicity of HP-Ag hydrogel on NIH/3T3 was evaluated using a CCK-8 assay in vitro, with PBS as the control group. As shown in Fig. 3A, after 12 h and 24 h culture, the relative cell growth rate of each group of hydrogels exceeded 86%, far higher than the minimum non-toxic standard of 70%. Similar results were obtained in proliferation experiments (Fig. 3B). There was no significant difference in cell proliferation rate between the HP-Ag hydrogel and control group, indicating that hydrogels had good cytocompatibility. After 3 days and 5 days of incubation, the cell activity of HP-Ag_M_ decreased slightly compared with PBS group, which may be due to the inactivation of some cells exposed to residual Ag NPs. Most conventional cross-linkers used in catechol polymers, such as Fe^3+^ and NaIO_4_, are cytotoxic, limiting their use as biomaterials. HP-Ag prepared using 4-arm PEG-SH and Ag NPs as cross-linkers had no cytotoxicity and is expected to be used in clinical wound dressings.

Hemocompatibility is an important factor to evaluate the compatibility between blood and biomaterials. The standard hemolysis activity test was used to evaluate the hemolysis ratio between homogeneous hydrogel and red blood cells (RBCs). As shown in Fig. 3C, hemolysis rates of HP and HP-Ag_M_ hydrogels were 1.83% and 1.13%, respectively. The hemolysis rates were similar to that of the PBS negative control, but much lower than that of the positive control and well below the allowable limit of 5%. Direct visual observation showed that the supernatant of the hydrogel group was yellowish and almost indistinguishable from the PBS group. In the positive group, the supernatant was bright red caused by RBCs lysis, indicating that the hemolysis of HP and HP-Ag_M_ hydrogels was negligible. The low hemolysis rate is due to the hydrophilicity of HA and 4-arm PEG-SH, which reduces RBCs destruction.

The antibacterial activity of HP-Ag hydrogel against gram-positive *S. aureus*, the representative cause of skin infections, was evaluated by colony counting. As shown in Fig. 3D, a large number of bacterial colonies appeared in the agar plate of PBS group, while no viable colonies appeared in HP-Ag groups, indicating strong bactericidal effects. Similarly, OD values of the S. aureus solutions co-cultured with HP-Ag hydrogel were significantly lower than that in PBS group, and the suspensions were clear after 12 and 24 h (Fig. 3E). These results suggest that HP-Ag hydrogel was effective in killing *S. aureus* and had negligible damage to cells. It should be emphasized that the highly effective antibacterial properties of HP-Ag hydrogel also greatly reduce the side effects of wound treatment that may be caused by high doses[40, 41]. As for possible antibacterial mechanisms, it is speculated that first the available phenol hydroxyl groups in HP-Ag hydrogel trap the bacteria, and then the positively charged Ag^+^ from Ag NPs interact electrostatic with the phosphoric groups of the phospholipids in the cell membrane, killing the bacteria by interfering with DNA replication and denaturing microbial proteins [42–44]. Overall, HP-Ag hydrogel showed superior cytocompatibility, hemocompatibility and strong antibacterial activity, which has great potential for clinical application.

### Accelerate wound healing process of acute wound

**Fig. 4:**
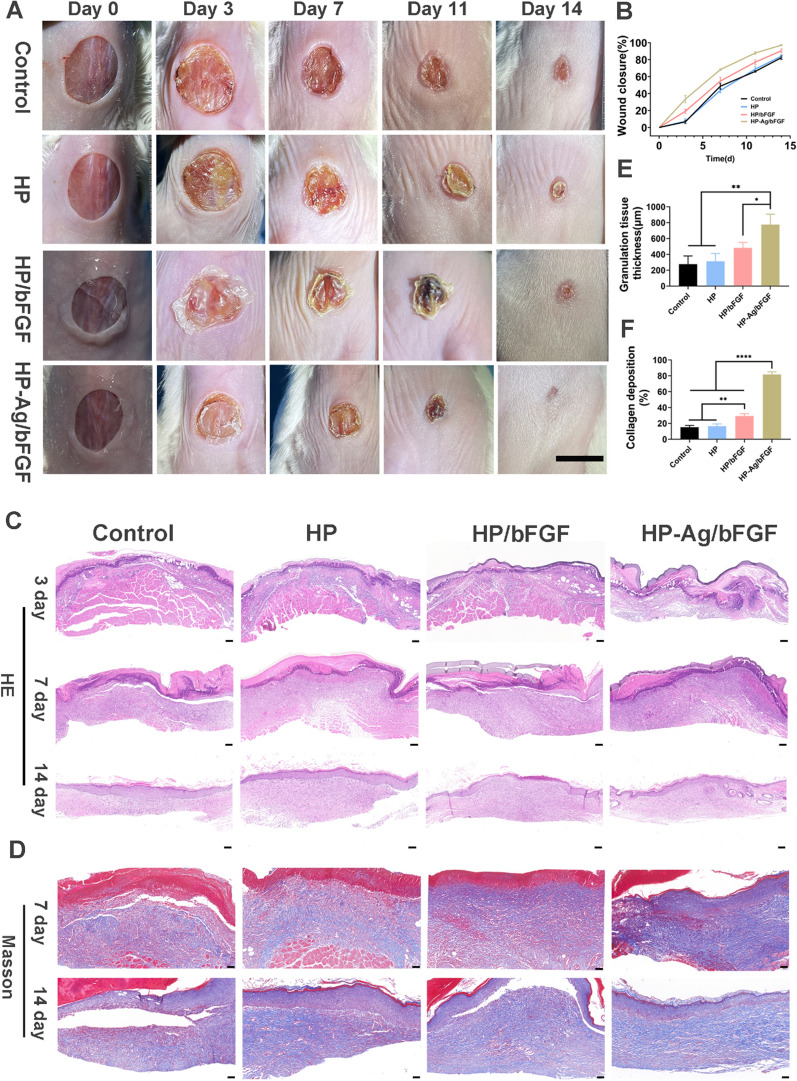
**A** Photographs of the skin wounds of various groups on day 0, 3, 7, 11and 14 (Scale: 10 mm). **B** Wound closure analysis from the existing wound area on day 3, 7, 11 and 14. **C** H&E staining of the wound sections on day 3, 7 and 14 (Scale: 100 μm). **D** Masson staining of the wound sections on day 7 and 14 (Scale: 50 μm). **E** Semi-quantification of skin granulation tissue thickness on day 7. **F** Collagen volume fraction of the wound sections on day 7. All data are presented as mean ± SD, *p < 0.05, **p < 0.01, ****p < 0.00001, n = 3

The wound healing processes of acute full-thickness skin defects were monitored photographically (Fig. 4A), and the corresponding calculation of the existing wound area were presented in Fig. 4B. The wound size of each group showed a decreasing trend over time. After 14 days of treatment, the wound area of HP-Ag/bFGF treatment was the smallest, and the wound became smooth and some new epidermal and dermal tissues appearing. In contrast, the skin surface of the wound in other groups was red with obvious scabs, suggesting the formation of early scar tissues. The redness of these scars is usually due to incomplete angiogenesis [19]. Wound closure varied significantly during the first 7 days after modeling due to self-contraction of the skin. The wound closure area of the control group (6.6%) was significantly lower than that of the HP-Ag/bFGF group (37.7%) on day 3 and was only 48.7% on day 7. The wound closure rate of HP-Ag /bFGF group reached 68.2%, higher than that of HP/bFGF group (54.7%). Compared with other groups, the HP-Ag/bFGF composite hydrogel group was more effective in promoting wound healing. On the one hand, the hydrogel formed in situ can fit the wound well and adhere closely to the wound site to avoid microbial infection. On the other hand, the water-retaining ability of HA preserves the moist environment required for wound healing, and HP-Ag/bFGF can sustainably provide bFGF for tissue regeneration.

In order to observe the process of wound healing more directly and accurately, the histological changes of skin were evaluated. H&E staining images (Fig. 4C) show that there was no or only a thin layer of granulation tissue under the skin of the control, HP and HP/bFGF groups, and a large number of inflammatory cells infiltrated. The semi-quantitative analysis (Fig. 4E) shows that the granulation tissue thickness in HP-Ag/bFGF group was 776 μm, which was obviously higher than that in other groups. On day 14, the new epidermis was obviously thickened and uneven in control group, and the inflammatory cells were obviously gathered under the epidermis, both of which could lead to the formation of scar at the wound site [45, 46]. However, the HP-Ag/bFGF group showed uniform epidermal tissue thickness, orderly granulation tissue, no evident inflammation occurred, and the regenerated dermis tissue with appendages like hair follicles and sebaceous glands was detected, all of which were important indicators of skin scarless healing.

As the support of cell growth, collagen can promote the proliferation and differentiation of tissue cells and create a better microenvironment. However, excessive production and disorderly deposition of collagen in the dermis can lead to scar production [5, 47]. Masson staining was used to evaluate collagen deposition at wound sites (Fig. 4D). The collagen deposition amount of HP-Ag/bFGF group was higher than that of other groups on day 7, and the collagen fibers were loosely and well-organized distributed in dermal tissue on day 14. This is consistent with the characteristics of scarless skin repair, where collagen accumulates in large quantities in the early stages and then partially fades and rearranges into the tissue [48]. Semi-quantitative analysis (Fig. 4F) shows that the collagen deposition was only 15.2% in control group on day 7, while it was 81.7% in HP-Ag/bFGF group, significantly higher than that in other groups. These results indicate that HP-Ag/bFGF composite hydrogels can accelerate wound regeneration and promote wound scarless healing.

TNF-α, a typical proinflammatory cytokine, was selected to evaluate the anti-inflammatory effect of hydrogels in vivo. The expression of TNF-α at the wound site on day 3 was shown in Additional file 1**:** Fig.S3 and Fig.S4. The expression of pro-inflammatory factors in control group was significantly higher than that in HP/bFGF and HP-Ag/bFGF group. In addition, TNF-α expression was lowest in HP-Ag/bFGF group, which was related to the continuous release of Ag to provide an anti-inflammatory micoenvironment. As shown in Additional file 1: Fig.S5, HP /bFGF and HP-Ag/bFGF group showed more mature blood vessels in the wound bed than the other groups. Long-term release of bFGF and Ag promotes the formation of mature blood vessels and the regression of immature blood vessels, thus providing adequate oxygen, nutrients, and growth factors for tissue regeneration.

Transforming growth factor TGF-β can induce fibroblasts to differentiate into myofibroblasts, which is closely related to scar formation in tissues. From Additional file 1: Fig.S6 and S7, the expression of TGF-β in HP-Ag/bFGF group was significantly lower than that in other groups. These results indicate that HP-Ag/bFGF composite hydrogel can facilitate scarless wound healing by down-regulating the expression of TGF-β.

### Evaluation of bacteria-infected wound healing in vivo

**Fig. 5:**
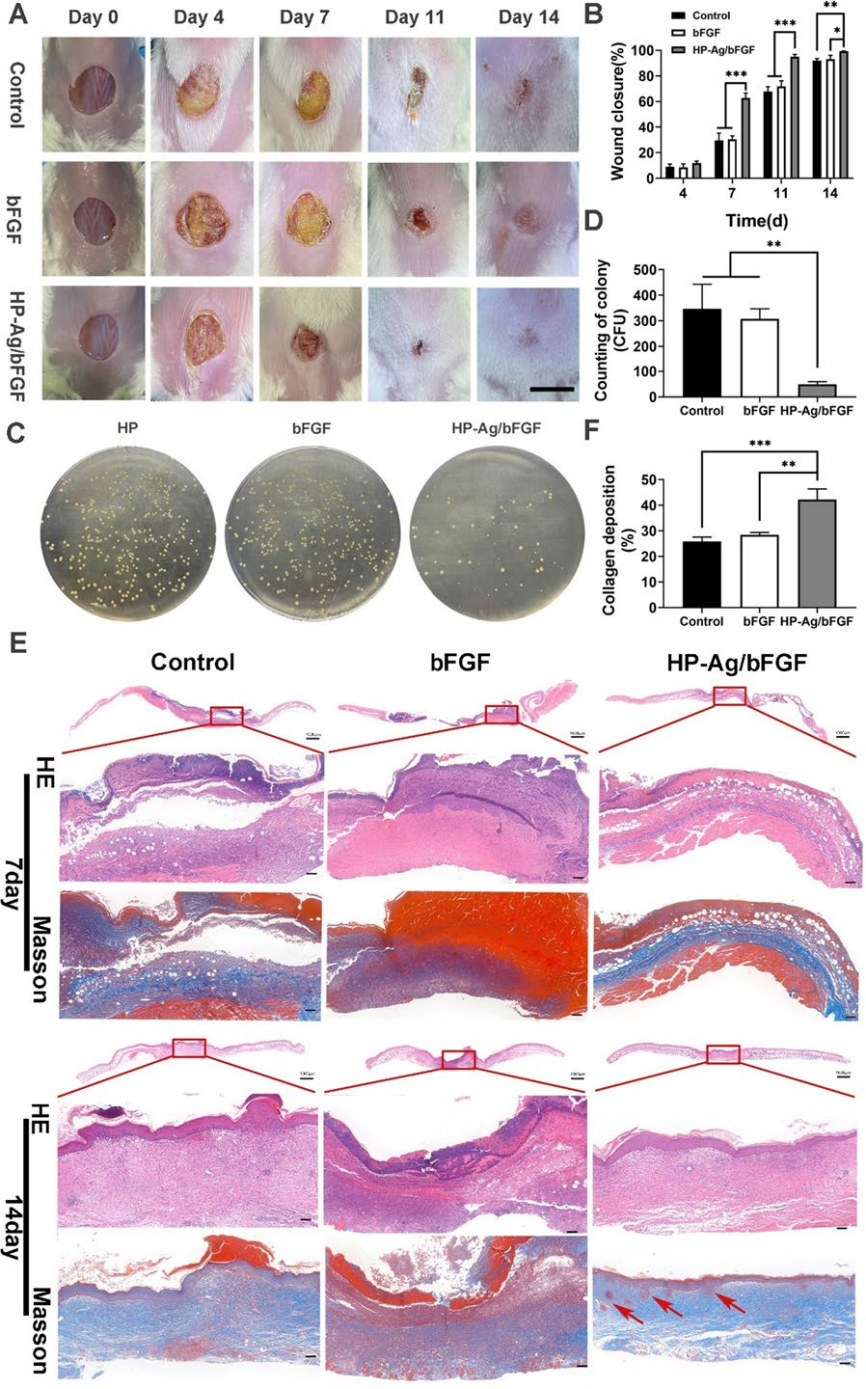
**A** Photographs of the skin wounds of various groups on day 0, 4, 7, 11and 14 (Scale: 10 mm). **B** Wound closure analysis from the existing wound area on day 4, 7, 11 and 14. **C** Photographs of survival bacteria colonies (*S. aureu*s, yellow dots in plates) growing on agar plates from wound sites on day 7 after different treatments. **D** Counting of survival bacteria colonies growing on agar plates from wound sites on day 7. **E** H&E and Masson staining of the wound sections on day 7 and 14. (Scale: 100 μm). **F** Collagen volume fraction of the wound sections on day 7 (red arrow: hair follicles). All data are presented as mean ± SD, **p < 0.01, ***p < 0.001, n = 3

The HP-Ag/bFGF hydrogel has the ability to continuously release Ag, and in vitro experiments have shown that it has a strong antibacterial effect. To further expand its clinical application, a bacterial infected wound model was used. Figure 5A shows that *S. aureus* at the wound site of the control group and the bFGF group grew wantonly. From day 4 to day 7, the wound was covered by a large number of bacterial colonies, which seriously hindered its closure process. There was no significant change in the rate of wound closure until the bacterial layer and blood scab were shed. However, the HP-Ag/bFGF group experienced a more regular closure process and was less affected by bacterial infection. On day 14, the wound was smooth and closed and even vanished for treatment group of HP-Ag/bFGF, whereas the wound boundaries were still observed for other groups. In the process of skin repair, the wound closure rate of HP-Ag/bFGF group was significantly higher than that of other groups (Fig. 5B).

On day 7, skin tissues were homogenized and diluted at an appropriate ratio before being smeared on the agar plate. Photographs and colony counts of infected skin in different groups are shown in Fig. 5C and D. The number of colonies in the HP-Ag/bFGF group was significantly less than that in the other two groups, which was consistent with the results of in vitro antibacterial experiments. These results confirm that HP-Ag/bFGF hydrogel was capable of destroying bacterial as an antibacterial platform, which is consistent with the results of previous studies [49].

As shown in Fig. 5E, similar to the acute wound, the epidermis in control group was thickened, with mastoid processes, and fibroblasts were abundant and irregularized. Meanwhile, the collagen fibers were numerous, dense and thick, with disordered arrangement, and the boundary between dermal reticular layer and epidermal layer was blurred. In contrast, the epidermal layer in HP-Ag/bFGF group was flat and thin, and there are fewer fibroblasts, and most of them are arranged in parallel. A key feature of optimal wound healing is remodeling the ECM by depositating collagen in a well-organized network to restore the normal structure of tissue. The loose and orderly arrangement of collagen and the presence of regenerated skin appendages such as hair follicles and sebaceous glands further confirm that HP-Ag/bFGF leads to scarless and effective regeneration. Collagen content in the HP-Ag/bFGF group was significantly higher than that in the other two groups on day 7, as shown in Fig. 5F.

**Fig. 6:**
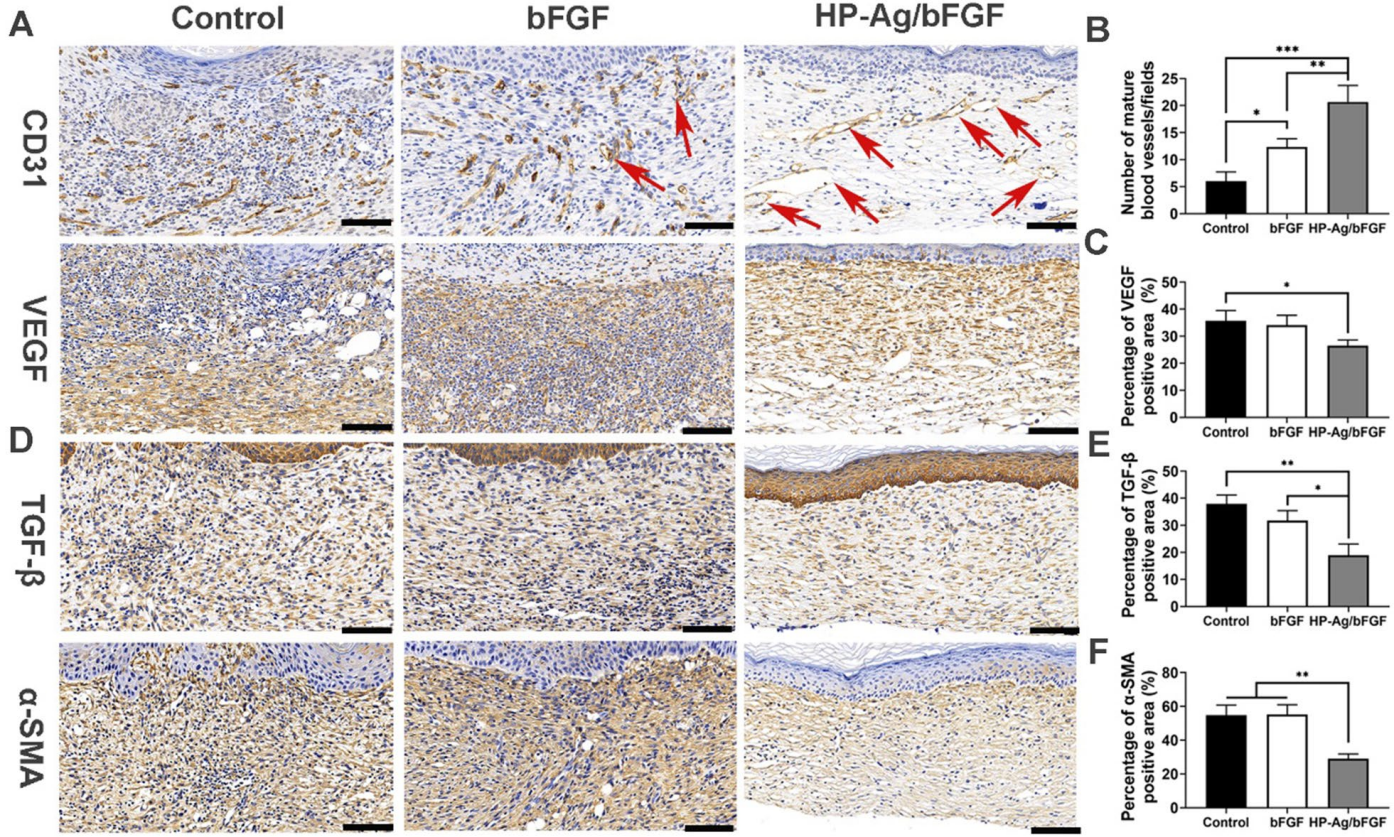
**A** Immunohistochemistry staining images of CD31(red arrow: mature blood vessels) and VEGF in the wound tissues on day 14. Semi-quantitative analysis of the expression level of CD31-positive ( +) mature vessels **B**, VEGF **C** in the wound tissues on day 14. **D** Immunohistochemistry staining images of TGF-β and α-SMA in the wound tissues on day 14. Semi-quantitative analysis of the expression level of TGF-β **E** and α-SMA **F** in the wound tissues on day 14 (Scale: 100 μm). All data are presented as mean ± SD. *p < 0.05, **p < 0.01, n = 3

Regeneration and remodeling of the blood supply system is critical for tissue regeneration. New blood vessels can provide necessary nutrients and oxygen supply for tissue reconstruction and carry away metabolic waste in time [50]. CD31 and VEGF were detected to evaluate wound angiogenesis. As shown by the CD 31 staining results in Fig. 6A, the immature capillaries in the control group were dense and disordered. Figure 6B and C show that HP-Ag/bFGF comprised significantly more mature vessels than the other two groups, while the temporarily constructed immature vessels degenerated over time. This control of vessel density by partially blocking capillary growth may lead to a reduced but fully functional vascular system, which has been shown to improve long-term healing outcomes and avoid scarring [51, 52].

TGF-β directly induces α-SMA expression in recruited fibroblasts, promoting myoblast differentiation into myoblasts, which in turn promotes uncontrolled ECM production, leading to scarring [53]. Therefore, TGF-β activity was associated with increased scar formation and fibrosis induction at the later stage of wound healing. Figure 6D and E show that the expression of TGF-β in HP-Ag/bFGF group was significantly less than that of the other two groups. These results suggest that HP-Ag/bFGF has greater potential to promote scarless wound healing. The low expression of α-SMA in Fig. 6F also confirmed this conclusion. In addition, as shown in Additional file 1**:** Fig.S8 and Fig.S9, the representative organs (heart, liver, spleen, lung, and kidney) were examined by H&E staining, and no obvious pathological abnormalities or damage were found in the organ sections, verifying the biosafety of HP-Ag/bFGF hydrogel for wound healing.

### Modulated macrophages polarization and inflammation response

**Fig. 7:**
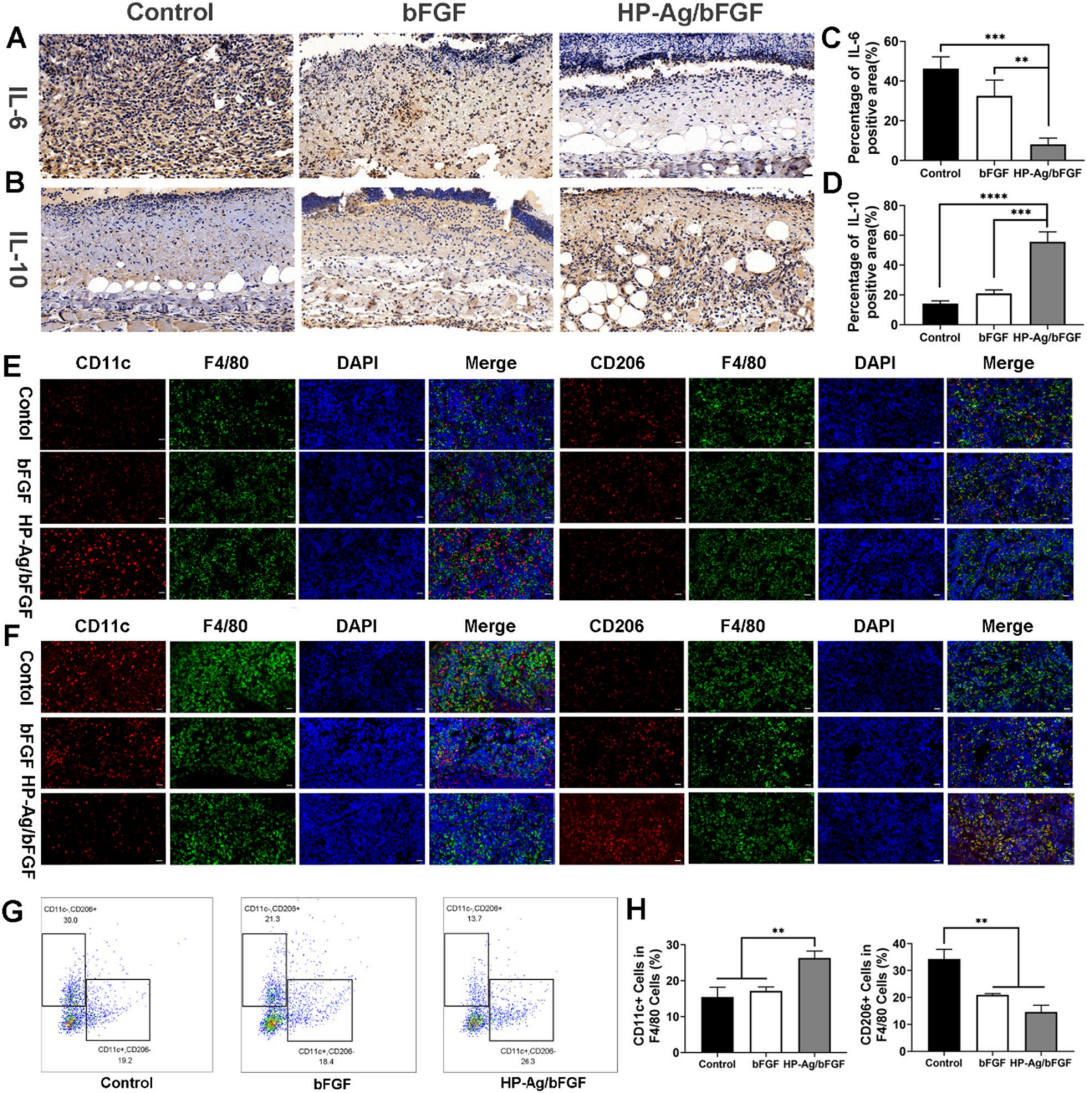
Regulation of inflammation and immune microenvironment. **A** Immunohistochemistry staining images of IL-6 in the wound tissues on day 4. **B** Immunohistochemistry staining images of IL-10 in the wound tissues on day 7. Semi-quantitative analysis of the expression level of IL-6 **C** and IL-10 **D**. Immunofluorescence staining of M1 and M2 type macrophage in the spleen of infected mice on day 4 **E** and day 7 **F**. Multicolor flow cytometry **G** and quantitative analysis **H** to detect the M1-type and M2-type macrophages in the spleen of infected mice on day 4. Scale bar: 20 μm (n = 3, **p < 0.01, ***p < 0.001, ****p < 0.0001)

Interleukin (IL) is a group of cytokines that play a crucial role in the inflammatory process. IL-6, a pro-inflammatory cytokine, is released early in inflammation and has been shown to be an effective stimulant of fibroblast proliferation [54]. IL-10, an anti-inflammatory and antifibrotic cytokine, has been reported to reduce the expression of IL-6, IL-8, and other inflammatory genes, and significantly improve collagen patterns [55, 56]. The content of IL-6 in tissues of HP-Ag/bFGF group was significantly lower than that in other groups, as shown in Fig. 7A and C. Meanwhile, the content of IL-10 in tissues of HP-Ag/bFGF group was significantly higher than that in other groups (Fig. 7B and D). These results suggest that compared with the other groups, the tissue inflammation was the weakest in HP-Ag/bFGF group on day 4 and the anti-inflammatory effect was the strongest on day 7.

Innate immunity is a crucial component of the body’s defense against pathogen invasion and plays a vital role in mediating tissue repair. The transformation of macrophages from M1 phenotype to M2 phenotype is believed to be necessary for wound healing [57]. On day 4 of acute wound infection, different macrophages subtypes were labeled by immunofluorescence and multicolor flow cytometry. M1-type macrophages (F4/80 + /CD11c +) are pro-inflammatory immune cells that secrete inflammatory cytokines during the acute phase of infection, while M2-type macrophages (F4/80 + /CD206 +) have anti-inflammatory and repair effects [58]. From Fig. 7E, the number of M1-type macrophages of HP-Ag/bFGF group was higher than that of the other two groups. Similarly, flow cytometry confirmed the results. As can be seen from Fig. 7G and H, M1-type macrophages in the HP-Ag/bFGF group were significantly higher than those in the other two groups, while M2-type macrophages were significantly lower than those in control group. The results indicate that the immune response in the control group and the bFGF group was relatively slow at the initial stage of wound infection, while the HP-Ag/bFGF group successfully stimulated the immune response through the release of Ag, accelerating the process of inflammation after infection and injury. Figure 7F shows the staining of M1-type and M2-type macrophages labeled by immunofluorescence 7 days after the infection. At this time, with the progress of tissue repair, the number of M2-type macrophages in HP-Ag/bFGF group was higher than that in other groups, while the number of M1-type macrophages was the lowest. The results indicate that the inflammatory period of the infected skin tissue in the HP-Ag/bFGF group had transitioned to the subsequent tissue repair period, while the control group and the bFGF group were still experiencing the inflammatory period. This observation is consistent with previous immunohistochemical results of IL-6 and IL-10.

## Conclusion

Based on both antibacterial and drug-carrying considerations, we proposed Ag NCH for the scarless healing of infected wounds. Ag NCH not only possesses good antimicrobial effects, but also has an enhanced network structure that enables the hydrogel to release bFGF and Ag/Ag^+^ on demand. Although the healing process differs slightly between acute wounds and infected wounds due to aspects such as the degree and duration of inflammation, both demonstrated that Ag NCH could promote skin wound regeneration and scarless healing. Specifically, Ag NCH was able to rapidly kill bacteria and promote macrophage polarization, thereby remodeling the wound microenvironment and accelerating the inflammatory process. In the later stages of wound regeneration, it regulates collagen deposition and accelerates the production of mature blood vessels, thereby promoting scarless wound healing. In conclusion, this study provides a therapeutic strategy for the clinical application for scarless healing of infected wounds.

### Supplementary Information

**Additional file 1:** **Figure S1.** Particle size distribution and zeta potential of Ag NPs.** Figure S2.** TGA curves of HA, HA-CA conjugates and HA-PEG hydrogel (A) and Ag NCH (B).** Figure S3.** Immunohistochemistry staining images of TNF-α in the wound tissues on day 3 (Scale: 100μm).** Figure S4.** Semi-quantitative analysis of the expression levels of TNF-α. All data are presented as mean ± SD. *p < 0.05, **p < 0.01, ***p < 0.001, n=3.** Figure S5.** Immunohistochemistry staining images of CD31 (red arrow: mature blood vessels) and VEGF in the wound tissues on day 14 (Scale: 100μm).** Figure S6.** Immunohistochemistry staining images of TGF-β in the wound tissues on day 14 (Scale: 100μm).** Figure S7.** Semi-quantitative analysis of the expression levels of TGF-β. All data are presented as mean ± SD. *p < 0.05, **p < 0.01, ***p < 0.001, n=3.** Figure S8.** H & E staining of main organs of acute wounds on day 14 (Scale: 50 μm).** Figure S9.** H & E staining of main organs of infected wounds on day14 (Scale: 50 μm).

## Data Availability

The datasets used and/or analyzed during the current study are available from the corresponding author on reasonable request.
