# Supplementary material for: Ag nanocomposite hydrogels with immune and regenerative microenvironment regulation promote scarless healing of infected wounds
Source: J Nanobiotechnology. 2023 Nov 19;21:435. doi: 10.1186/s12951-023-02209-2 (PMC10658971; doi:10.1186/s12951-023-02209-2)
Supplement: Supplementary file 1 — Additional file 1: Figure S1. Particle size distribution and zeta potential of Ag NPs. Figure S2. TGA curves of HA, HA-CA conjugates and HA-PEG hydrogel (A) and Ag NCH (B). Figure S3. Immunohistochemistry staining images of TNF-α in the wound tissues on day 3 (Scale: 100μm). Figure S4. Semi-quantitative analysis of the expression levels of TNF-α. All data are presented as mean ± SD. *p < 0.05, **p < 0.01, ***p < 0.001, n=3. Figure S5. Immunohistochemistry staining images of CD31 (red arrow: mature blood vessels) and VEGF in the wound tissues on day 14 (Scale: 100μm). Figure S6. Immunohistochemistry staining images of TGF-β in the wound tissues on day 14 (Scale: 100μm). Figure S7. Semi-quantitative analysis of the expression levels of TGF-β. All data are presented as mean ± SD. *p < 0.05, **p < 0.01, ***p < 0.001, n=3. Figure S8. H & E staining of main organs of acute wounds on day 14 (Scale: 50 μm). Figure S9. H & E staining of main organs of infected wounds on day14 (Scale: 50 μm). [file 12951_2023_2209_MOESM1_ESM.docx]

Additional file information

Ag nanocomposite hydrogels with immune and regenerative microenvironment regulation promote scarless healing of infected wounds

Yihui Zhang, Jian Kang, Xuan Chen, Wenkai Zhang, Xiangqi Zhang, Wei Yu, Wei-En Yuan*


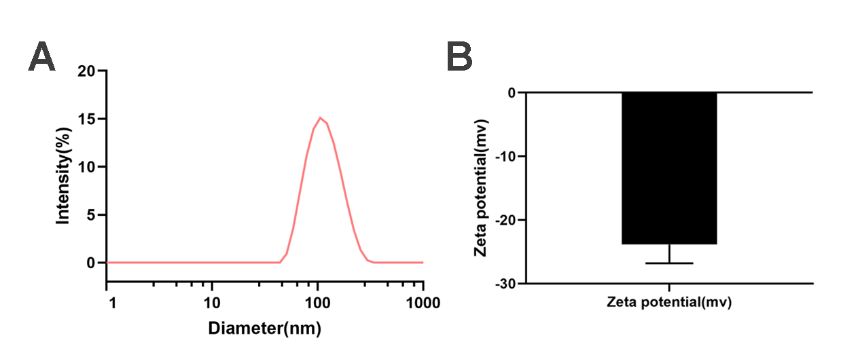


Figure.S1 Particle size distribution and zeta potential of Ag NPs.

­­
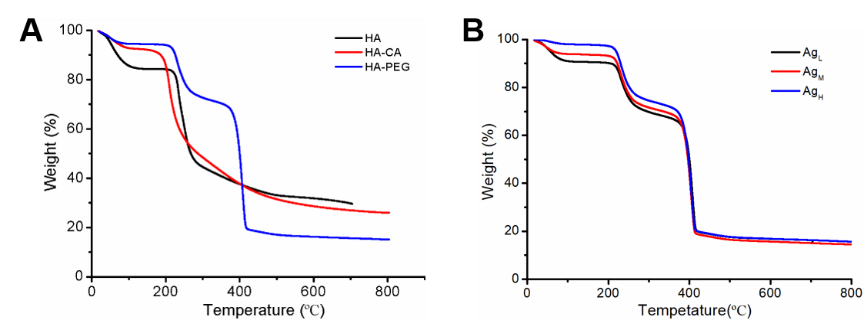


Figure.S2 TGA curves of HA, HA-CA conjugates and HA-PEG hydrogel (A) and Ag NCH (B).


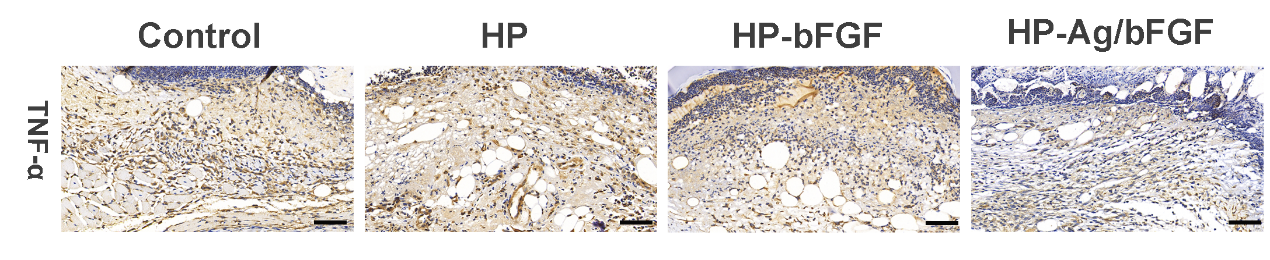


Figure.S3. Immunohistochemistry staining images of TNF-α in the wound tissues on day 3 (Scale: 100μm).


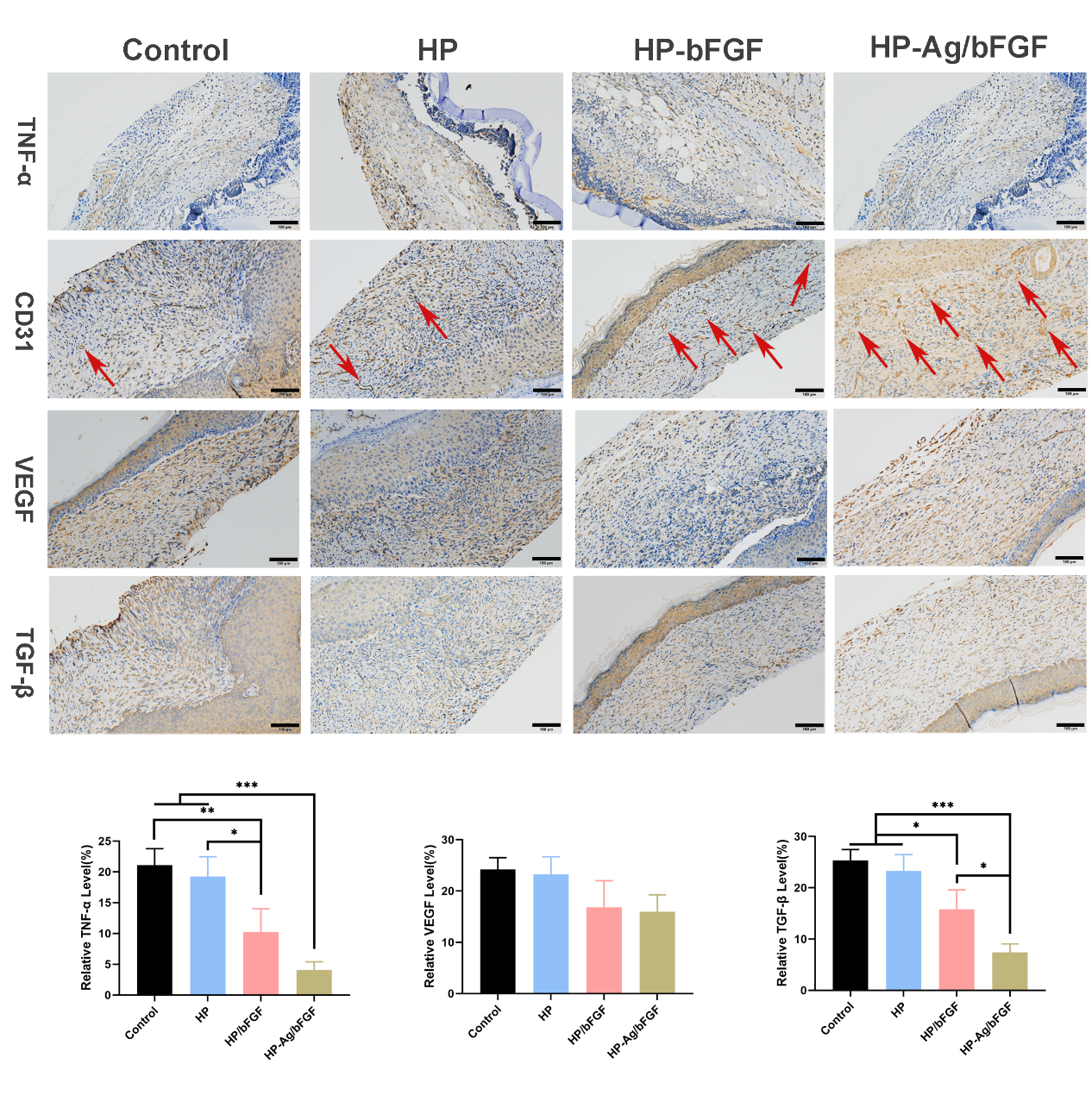


Figure.S4. Semi-quantitative analysis of the expression levels of TNF-α. All data are presented as mean ± SD. *p < 0.05, **p < 0.01, ***p < 0.001, n=3.


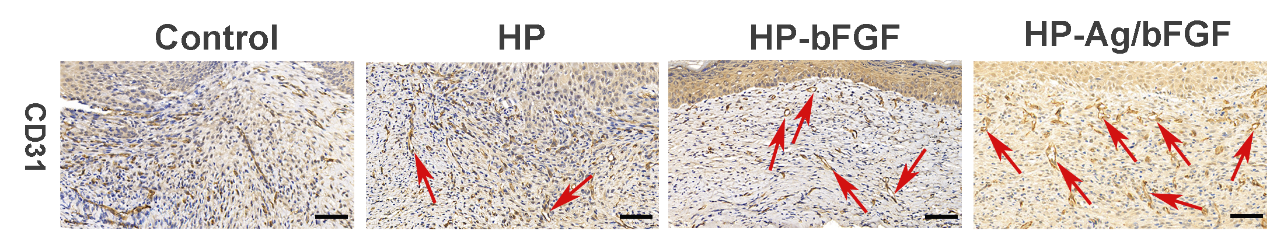


Figure.S5. Immunohistochemistry staining images of CD31 (red arrow: mature blood vessels) and VEGF in the wound tissues on day 14 (Scale: 100μm).


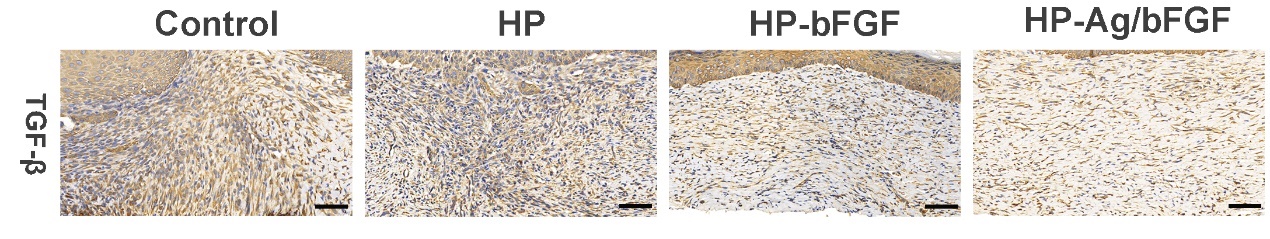


Figure.S6. Immunohistochemistry staining images of TGF-β in the wound tissues on day 14 (Scale: 100μm).


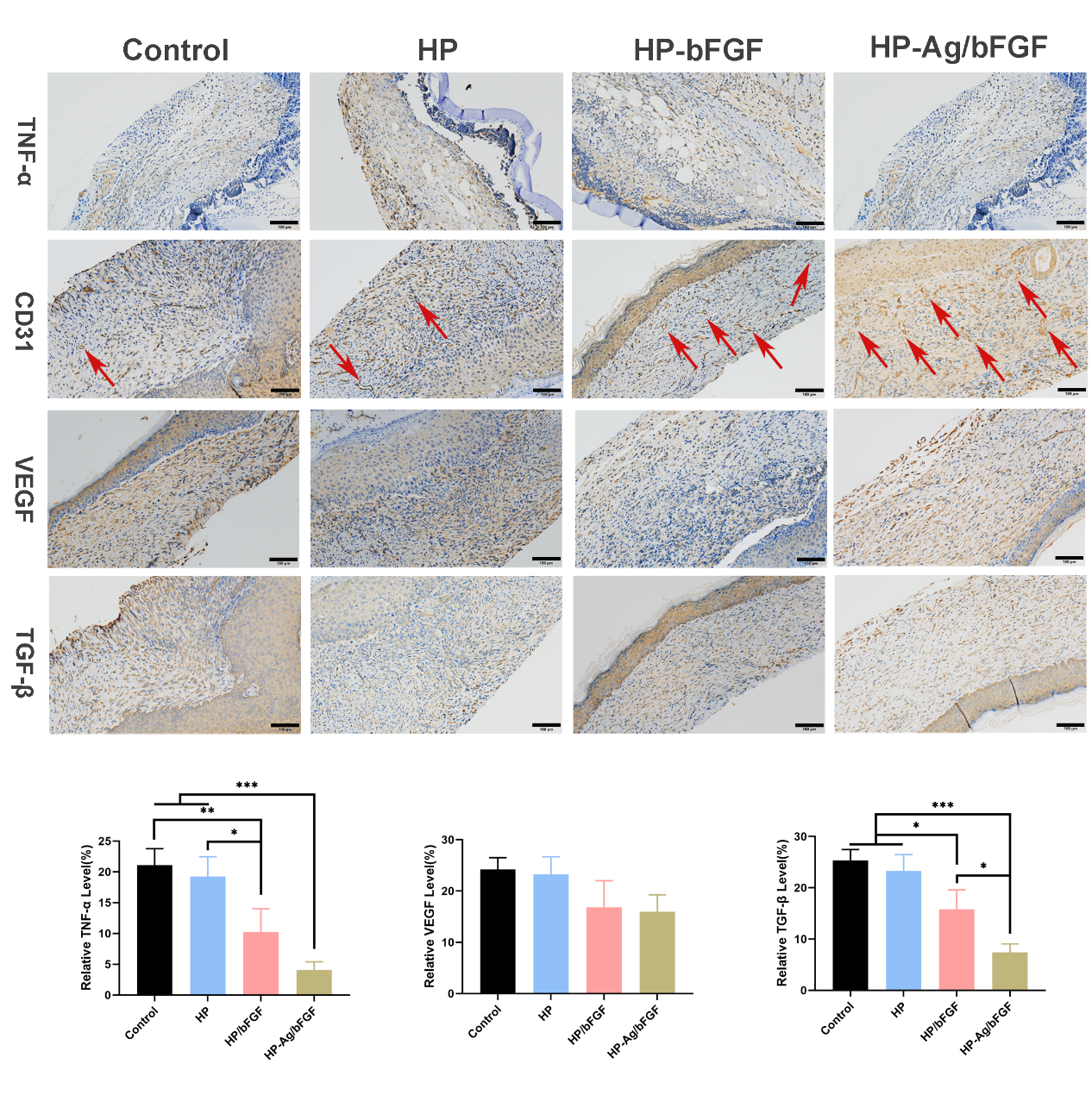


Figure.S7. Semi-quantitative analysis of the expression levels of TGF-β. All data are presented as mean ± SD. *p < 0.05, **p < 0.01, ***p < 0.001, n=3.


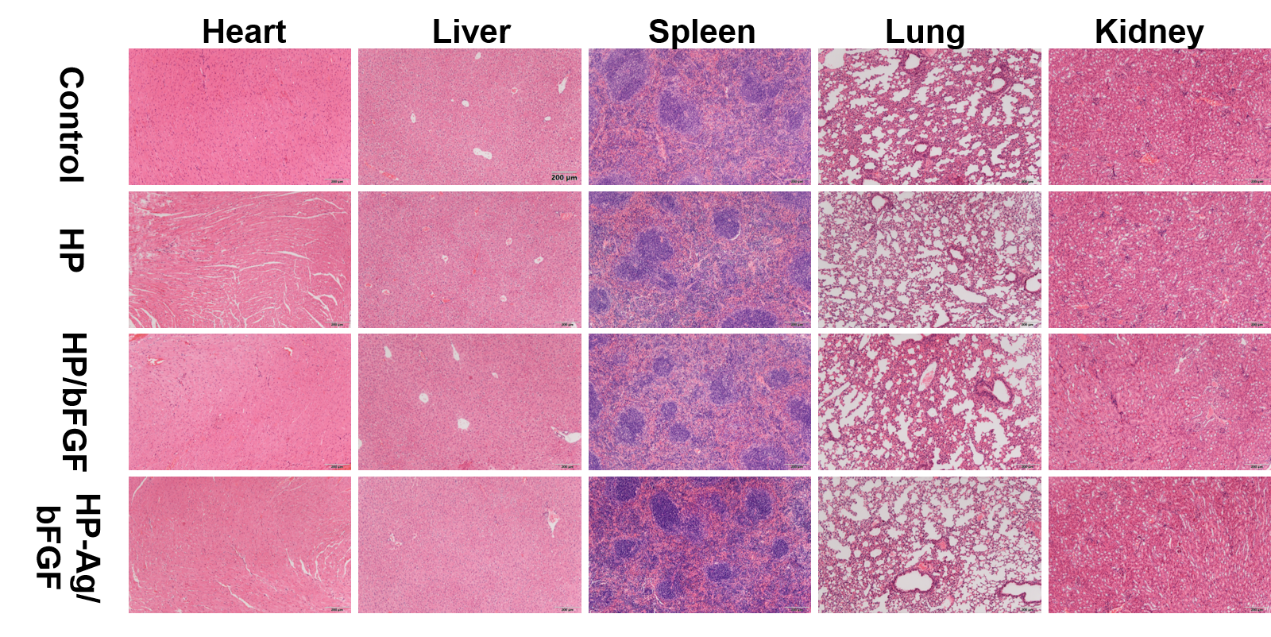


Figure.S8. HE staining of main organs of acute wounds on day 14 (Scale: 50 μm).


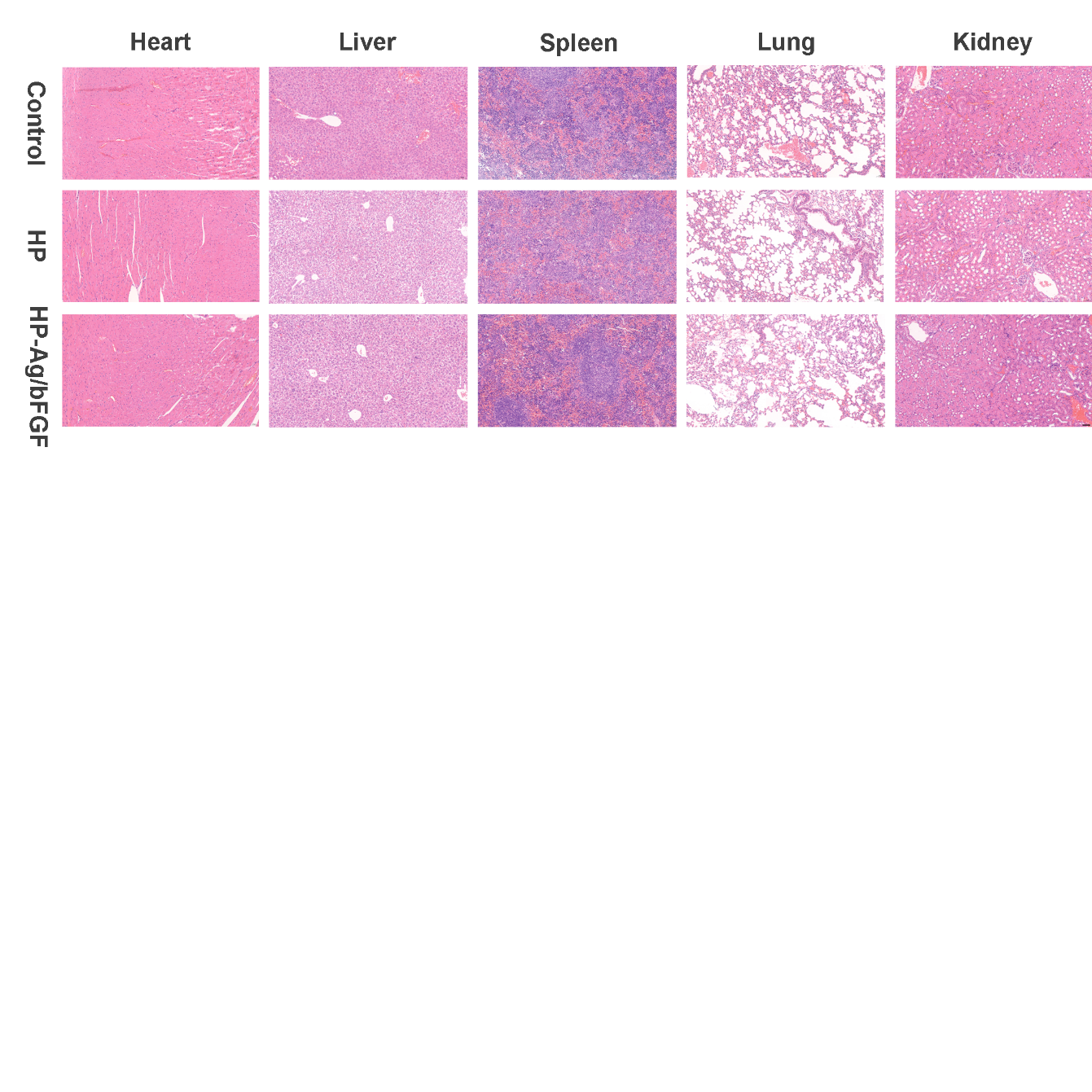


Figure.S9. HE staining of main organs of infected wounds on day14 (Scale: 50 μm).
